# The new COSMIN guidelines confront traditional concepts of responsiveness

**DOI:** 10.1186/1471-2288-11-152

**Published:** 2011-11-18

**Authors:** Felix Angst

**Affiliations:** 1 Research Department, RehaClinic Zurzach, 5300 Bad Zurzach, Switzerland; 2 Department for Upper Extremity and Hand Surgery, Schulthess Klinik, 8008 Zurich, Switzerland

## Abstract

The recently published "COSMIN" guidelines aim to rate properties of outcome instruments and state two issues with regard to responsiveness which is the instrument's ability to detect change over time. These issues are comparison of score changes with change of an external criterion using correlations and the judgement of traditional methods as inappropriate. The latter are the "transition" concept, a global rating of change, and parametric measures of responsiveness, for example, effect sizes. It can be shown that the methodology proposed by the guidelines has important weaknesses and that denunciation of traditional methods is not appropriate. Some claims of the guidelines about responsiveness do not match the demands of clinical reality and confront findings of numerous epidemiological studies.

## Instructions and demands of the COSMIN guidelines

At the department of epidemiology and biostatistics, University Medical Center, Amsterdam, NL, a group recently established guidelines to assess and prove measurement properties and qualities of self-assessed health outcome instruments [1,2]. The criteria for the guidelines were established using the Delphi method [1]. However, it can be shown in the following that substantial methods of methodological or empirical literature were not consulted. For this reason, it is likely that a debate on the conclusions and instructions will be raised. In this COSMIN (COnsensus-based Standards for selection of health Measurement INstruments) checklist manual, the methodology to examine responsiveness, i.e. "the instrument's ability to detect change over time in the construct to be measured" gives the following instructions [1,2]:

Score changes over time as derived from the assessment instruments should be compared and correlated to those of a "gold-standard" or an "external criterion", see p. 5 of [2]. If no gold-standard is available the score changes of an instrument under examination have to be correlated to "changes in other variables, such as scores on other instruments, or demographic or clinical variables", see items 8-14, p.40/41 of [1]. This should be done by hypotheses formulated a priori about the direction and magnitude of the score change correlations [1,2]. This means that direct comparison of the score changes of two or more instruments, as has been done according to traditional concepts to date, is obsolete. In particular, parameters like effect sizes (ES), standardized response means (SRM), or Guyatt's responsiveness ratio, and other well known parameters in common use are "inappropriate" and should not be used to assess responsiveness, see p. 42 of [1] and p. 6 of [2].

## The issue of gold-standard or external criterion

With regard to these requirements and claims, the following comments can be made. In most cases of empirical research, neither a "gold-standard" nor an "external criterion" exists. One example for that is the measurement of pain since pain is a complex bio-psycho-social sensation and is especially individual in terms of perception, processes, and coping. Although the COSMIN authors admit that a gold-standard is generally impossible to find, they do not offer a solution for this problem, see items 15-18, p. 42 of [1].

On p. 40 of the manual, it is stated that "sometimes, a global rating scale of change was used to ask patients if they considered themselves as changed on the construct" - remark: to obtain an external criterion [1]. The question about the global rating of change is the well-known "transition" question. The concept was introduced in 1989 by Jaeschke et al. [3] and has been used in numerous studies.

However, on p. 42 of the manual, it is stated: "some authors have questioned the reliability and validity of such retrospective measures of change [4]. Therefore, this rating scale was not considered to be an appropriate gold standard for assessing responsiveness. It could, however, be considered a useful comparator instrument in a construct approach" [1]. It remains unclear for the reader whether the transition method is appropriate in the absence of a gold-standard. If not this implies that research over 20 years and hundreds of published studies that used the transition concept would become irrelevant. Although the transition concept may have its shortcomings it is the only one that is available to solve this problem and it has been applied up to now despite the criticism.

Responsiveness aims at detecting change over time in the construct to be measured. When comparing instrument 1 to instrument 2, the more responsive instrument is more likely to detect changes over time than the other one. Taking an example, we only want to know whether camera 1 takes sharper/better contrasted pictures (over time: as a film) than camera 2 to detect objects. Two articles that tried to explain the COSMIN rules demand that, in this case, the score change of instrument 1 (= a) must be compared to that of an external criterion/gold standard (= c) and then be related to the change of instrument 2 (= b); see p. 5 of [2] and the empirical example [5]. The differences of these score changes to the score change of the external criterion (c) will be (a-c) and (b-c). The difference between these differences is (a-c)-(b-c) = a-b by the well-know mathematical translation rule. Analogously, the correlation of (a-c) to (b-c) is the correlation of a to b. In both cases, the external criterion and its score change (c) is not necessary. Likewise, what the picture/objects are like in physical "reality" is not important - nobody knows it - because our eye plus our optical cortex depict only a part of the physical reality (= the external criterion) and this varies from individual to individual.

## The issue of comparing responsiveness by correlations

Further insight into the COSMIN concept is given by an exemplary study on how to assess responsiveness [5]. In this study, a number of predefined hypotheses were stated to test responsiveness/longitudinal construct validity. The hypotheses compare correlations of score changes between instruments. For example: "Correlation of change on instrument 1/instrument 2 with change on instrument 3 (= the first external criterion) is higher than correlation with the global rating question (= the transition question as second external criterion) by (an expected correlation difference of) 0.1"; see table 2 of [5] or p. 5 of [2]. In this example, 8 hypotheses were stated for mobility of which 6 (75%) were refuted. This was interpreted as poor responsiveness. 25-50% refuted hypotheses means moderate, < 25% high responsiveness.

This example led to the following comments: The kind, content, and number of the hypotheses are arbitrary. Comparing two instruments, the list of possible hypotheses is almost indefinite depending on how much detail is sought. This means that the proportion of refuted hypotheses is arbitrary and is dependent on the moment when the stating of hypotheses stopped. This proportion is the basis for the categorized rating of responsiveness, e.g. > 50% refuted hypotheses means poor responsiveness. First, these thresholds are also arbitrary and not empirically validated. Second, the expected correlation difference of 0.1 is arbitrary, not empirically validated and has no clinical importance. These two problems are even explicitly stated in the discussion of the exemplary study [5].

Verification and falsification of hypotheses is only a qualitative, not a quantitative analysis as given by the concept of ES or SRM. As a hypothetical example (to the exemplary study) case 1 is that (score change) correlation of the first instrument to the external criterion is 0.60 and 0.71 of the second instrument - the correlation difference is 0.11. Case 2 is that the correlation of the first instrument is 0.01 and of the second instrument is 0.99 - the correlation difference is 0.98. In both cases, the hypothesis, namely, the correlations' difference of 0.10 is true but the different amount of the correlation differences is not relevant when using the COSMIN concept as done in [5]. In addition, the exemplary study which aimed to explain the guidelines made use of the transition concept as an external criterion [5]. But the transition concept has been refused as an external criterion in the guidelines [1]. Finally, instrument 3 in that study was a questionnaire which had not been longitudinally validated according to the COSMIN principles before and can, consequently, not be used as a valid external criterion [5].

## The issue of traditional responsiveness measures

Responsiveness parameters such as ES, SRM etc. are accepted worldwide and used in a large body of scientific literature as been shown below. Nowhere in the manual do the authors give a detailed explanation or arguments as to why these parameters are "inappropriate"; see p. 42 of [1] and p. 6,7 of [2]. The reader may find an implicit explanation in the statement that "these measures are considered measures of the magnitude of an intervention or the event, rather than measures of the quality of the measurement instrument", see p. 42 of [1] and p. 6 of [2].

Traditional responsiveness studies administer two measures at the same time points, in the same situation, and to the same patients. Therefore, the "intervention" and the "event" must be the same for both instruments and differences of score changes, i.e. differences between ESs and SRMs, must be caused by the differences in the quality of the two instruments.

As a second reasoning can be found in the exemplary study on p. 234 [5]: "Measures of treatment effects such as effect sizes of the t-test statistic are in itself only useful for interpretation of score changes, not for assessing the responsiveness of a measure, because it will not be possible to infer if a corresponding change in the concept has taken place". However, it is not further explained what "change in the concept" could take place or what "infer" means in this context and the sence of this statement remains unclear for the reader.

The concept of measuring change over time by ES (= mean score change divided by standard deviation at baseline) and related parameters was introduced by Rosenthal and Kazis for the interpretation of changes in health status as a result of decades of previous research [6,7]. In particular, it has also been used to compare responsiveness of two instruments. A short Pubmed review (on 25/2/2011 with keywords: responsiveness and (ES or SRM) reveals that the application of this concept has been reported in 1,387 research articles. To label that as "inappropriate" means to confront the rest of the research world and to set aside the research efforts and publications of decades. Moreover, all ongoing studies designed on the basis of these concepts and assessment strategies will not be able to publish their future responsiveness results. All currently used instruments that involve testing of responsiveness by quantitative measures such as ES, SRM etc. could no longer be considered valid and all clinical studies that utilized these instruments would have invalid results.

With regard to Guyatt's responsiveness ratio being an "inappropriate" measure, some of the members of the COSMIN group, namely Terwee et al., published contrary statements in their paper entitled "Quality criteria were proposed for measurement properties" in 2007 [8]: "Responsiveness should therefore be tested by relating the smallest detectable change to the minimal important change. This approach is equalent to the Guyatt's responsiveness ratio, ...".

## Conclusions and implications for clinical practice

Clinical practice is confronted with the difficulty that clinicians need to be able to comprehend and apply the concepts of responsiveness of assessment instruments in daily routine. ES and SRM are heuristic measures of change and have found acceptance in science and clinical practice over many years. The quantitative amount of change is important for the clinician and should be easy to comprehend. To obtain an overview and assess proportions of correlation differences as proposed by the COSMIN guidelines is difficult and not readily accessible. It can be expected that many clinicians may have difficulty understanding and applying this concept and the methodology will be limited to a small circle of researchers. Dogmatic introduction of the COSMIN responsiveness guidelines at once, without transition and without acceptance of concurrent concepts is likely to result in low acceptance and marginal clinical importance.

In summary, standardized rules to test instruments are welcome in research. The COSMIN rules to examine responsiveness leave many open questions. How can we deal with the problems and contradictions described above? It should be possible to reassess the "inappropriateness" of the transition concept and of the parametric measures (ES, SRM etc.) through detailed and balanced arguments. The COSMIN concepts may have a supplementary value when focussing on longitudinal construct validity. For most clinicians, responsiveness is not (only) a question of longitudinal validity - they simply wish to find that instrument that more accuately detects changes over time than the other by a quantitative measure - not more and not less. It should be possible to take the positive properties of both concepts, the traditional and those of COSMIN, to find improved standards and to proceed according to the principle of "live and let live". If not, how shall we deal with the validity and responsiveness findings of existing literature, research, and clinical routine?

## Competing interests

The author declares that they have no competing interests.

## Authors' response

### The new COSMIN guidelines regarding responsiveness

Lidwine B Mokkink, Caroline B Terwee, Dirk L Knol, Henrica CW de Vet

Address: Department of Epidemiology and Biostatistics and the EMGO Institute for Health and Care Research, VU University Medical Center, Amsterdam, the Netherlands

Emails:

LBM: http://w.mokkink@vumc.nl

CBT: http://cb.terwee@vumc.nl

DLK: http://d.knol@vumc.nl

HCWV: http://hcw.devet@vumc.nl

In this piece, Dr Angst argues that some of the claims of the COSMIN guidelines about responsiveness do not match the demands of clinical reality and confront findings of numerous epidemiological studies.

We thank Dr Angst for his interest in the COSMIN checklist and we think he raises some relevant issues concerning responsiveness. Before we give our reaction to these issues, we would like to emphasize that the COSMIN checklist was developed to evaluate the methodological quality of studies on measurement properties. It is not a checklist to assess the quality of a measurement instrument. Furthermore, we think it is important to mention that the COSMIN checklist was developed in a Delphi study in which over 40 international experts were involved. The members of the Steering Committee did not have a vote in these Delphi rounds [9].

We do not agree with all issues raised by Angst. However, we might not have been clear enough in our manual. Therefore, we would like to take the opportunity to explain the viewpoints of COSMIN regarding responsiveness in more detail. Based on the remarks of dr Angst we further clarified some issues in the COSMIN manual [1].

In our response, we focus on four points which will elucidate COSMIN's ideas around responsiveness and deal with the concerns of dr Angst:

Responsiveness is longitudinal validity and therefore the assessment of responsiveness closely follows the way in which validity of measurement instruments is assessed.

A distinction should be made between the interpretation of changes in health status and responsiveness as a measurement property of a measurement instrument.

The literature on responsiveness using effect sizes and other "inappropriate measures" should not be thrown away, but provides less evidence than previously thought.

The COSMIN guidelines do not reject the "transition" question, but recommend to test hypotheses about expected relations with the "transition" question.

### Responsiveness is longitudinal validity

Dr Angst argues that "For most clinicians, responsiveness is not (only) a question of longitudinal validity - they simply wish to find that instrument that more accurately detects changes over time than the other by a quantitative measure". According to COSMIN, this is the same because accurate detection of change means measuring the true amount of change, which is a matter of longitudinal validity.

The COSMIN panel was very clear that responsiveness should be considered as longitudinal validity. If you want to measure change, a valid instrument should truly measure changes in the construct(s) it purports to measure. The only distinction between (construct and criterion) validity and responsiveness is that validity concerns the validity of single scores while responsiveness concerns the validity of change scores. Consequently, the COSMIN panel concluded that responsiveness should be evaluated similarly as validity, i.e. by comparing changes on the instrument with changes on the gold standard, or - since often there is no gold standard - by testing hypotheses e.g. about expected correlations with changes in other measures, or expected differences in changes between groups.

One of the most difficult tasks when testing hypothesis, is formulating challenging hypotheses. By testing hypotheses we aim to show that the instrument truly measures changes in the construct(s) it purports to measure. In practice, this means that the instrument should measure (changes in) the right construct(s) and not (changes in) something else, but also that it should measure the right amount of change, i.e. it should not under- or overestimate the real change in the construct that has occurred. This latter aspect is often overlooked in assessing responsiveness. In the COSMIN manual we explain that specific hypotheses should therefore include an expectation about the direction and magnitude of the correlation between changes in the instrument under study and changes in a comparator instrument, or an expectation about differences in change scores on the instrument between groups.

In one of the COSMIN articles [2], we provided some examples of hypotheses based on one of our previous studies [5]. We would like to emphasize that these hypotheses were only used as examples. The COSMIN panel considered it not possible to formulate standards for the amount of hypotheses that need to be tested in a construct validity study. This depends on the construct to be measured and the content and measurement properties of the comparator instruments [1]. The definition of criteria for good measurement properties was beyond the scope of the COSMIN study.

Note that also for assessing validity we also have no quantitative measures and we also test an arbitrary chosen number of hypotheses. There is no criterion to decide whether an instrument is valid or responsive. Assessing validity or responsiveness is a continuous process of accumulating evidence.

### Distinction between the interpretation of changes in health status and responsiveness as a measurement property of a measurement instrument

Effect sizes and related parameters have been introduced by Cohen [10] to provide a standardized measure of the magnitude of an effect. These measures are used to interpret changes in health status, or magnitudes of treatment effects.

It is impossible to assess in one study both the treatment effect and the responsiveness of measurement instrument based on the same effect size. If the effect size is zero, either the intervention has no effect or the outcome measure is not responsive. If the effect size is moderate, more conclusions are possible: either the effect is moderate and the outcome measure is responsive, or the effect is large or small and the outcome measure has poor responsiveness because the true effect is over- of underestimated by the instrument. So the argument of the COSMIN panel is that the effect size only has meaning as a measure of responsiveness if we know (or assume) beforehand what the magnitude of the effect of the intervention is. If, for example, we expect a large effect of the intervention we can test the hypothesis that the measurement instrument shows an effect size of 0.8 or higher. But if we expect a small effect of the intervention, we would not expect such a high effect size. This example shows that a high effect size does not necessarily indicates a good responsiveness.

When several instruments are compared in the same study, this could give evidence for the relative responsiveness of the instruments. But again, only if a hypothesis is being tested including the expected magnitude of the treatment effect. Let us propose that we have three measurement instruments (A, B, and C), all measuring the same construct. The intervention given is expected to moderately affect the construct measured by the three instruments. Results show that instrument A has an effect size of 0.8, instrument B of 0.40 and instrument C of 0.15. Based on our hypothesis of a moderate effect we should conclude that instrument B appears to best measure the construct of interest. Instrument A seems to over-estimates the treatment effect (e.g. because it shows change in persons who do not really change), and instrument A seems to under-estimates it. This example shows that it may not always be appropriate to conclude that the instrument with the highest effect size is the most responsive.

### The literature on responsiveness using effect sizes and other "inappropriate measures" should not be thrown away, but provides less evidence than previously thought

In the previous paragraphs, we have tried to explain that the COSMIN panel does not totally discard effect sizes as parameters of responsiveness, but argues that it is necessary to formulate and test hypotheses about the magnitude of change that is to be expected from the treatment. Many responsiveness studies, however, have been published (and still are) in which an instrument was considered responsive just because the effect size was larger than 0.8. This is what COSMIN considers inappropriate.

Note that COSMIN does not intend to set aside decades of research. Published studies do provide evidence for responsiveness, but less than previously thought. These studies can be included in systematic reviews of measurement properties. However, it is then up to the authors of the review to decide (in retrospect) whether the results found are as could have been expected, taking the treatment, the construct to be measured, the population etc. into account. This may, however, be more difficult and more prone to bias than formulating hypotheses before the data collection.

### The COSMIN guidelines do not reject the "transition" question, but recommend to test hypotheses about expected relations with the "transition" question

We agree with dr Angst that there is no gold standard for pain and other PROs measuring symptoms and perceptions and that the "transition" question (global rating of change) is often the only criterion available for measuring change in PROs. However, there is an ongoing debate on its validity and reliability [11,12]. It is therefore still unclear whether the "transition" question should be considered a gold, silver, or no standard at all. Therefore the COSMIN panel proposed to consider it a construct approach of responsiveness (comparable to construct validity) if the "transition" question is used to assess responsiveness. In that case it is recommended to define and test hypotheses e.g. about the expected correlation between changes on the instrument under study and the "transition' question. Moreover, it should not be the one and only hypothesis to be tested. This is what we presented in our example.

Similar as with effect sizes, COSMIN does not discard the use of a "transition" question in the assessment of responsiveness, but recommends to formulate and test hypotheses about what correlations are to be expected.

## Conclusion

We agree with dr Angst that the quantitative amount of change is important for the clinician and should be easy to comprehend. Therefore, the aim of a responsiveness study is to show that the instrument indeed measures the true amount of change that has occurred in the construct of interest. According to the COSMIN panel, the best design to show this is by testing hypotheses about expected correlations or changes. Effect sizes and "transition" questions can be used, but they only provide evidence for responsiveness if clear hypotheses about expected changes are being tested.

The COSMIN approach for assessing responsiveness is not more difficult than assessing validity. In fact, the COSMIN panel proposed exactly the same approach because responsiveness is defined as longitudinal validity.

We acknowledge that the COSMIN panel has set high standards. The main reason to do so is to improve future studies on measurement properties, and to challenge readers of published studies to be critical when interpreting results. It is not our intention to make all research of the past 20 or 30 years irrelevant. We only want to emphasize that because of new insights, we could do better.

## Pre-publication history

The pre-publication history for this paper can be accessed here:

http://www.biomedcentral.com/1471-2288/11/152/prepub
